# Pulmonary edema following subarachnoid hemorrhage is associated with impairment of pulmonary vascular endothelial glycocalyx

**DOI:** 10.1016/j.bbrep.2025.102420

**Published:** 2025-12-18

**Authors:** Nozomi Sasaki, Yusuke Egashira, Hideshi Okada, Chihiro Takada, Shinomi Sasaibe, Masaki Kumagai, Yoshiki Kuse, Shinsuke Nakamura, Hirofumi Matsubara, Yukiko Enomoto, Toru Iwama, Tsuyoshi Izumo, Hideaki Hara, Masamitsu Shimazawa

**Affiliations:** a Department of Neurosurgery, Gifu University Graduate School of Medicine, Gifu, Japan; b Molecular Pharmacology, Department of Biofunctional Evaluation, Gifu Pharmaceutical University, Gifu, Japan; c Laboratory of Collaborative Research for Innovative Drug Discovery, Gifu Pharmaceutical University, Gifu, Japan; d Department of Emergency and Disaster Medicine, Gifu, University Graduate School of Medicine, Gifu, Japan; e Department of Neurosurgery, Gifu Municipal Hospital, Gifu, Japan; f Department of Neurosurgery, Takayama Red Cross Hospital, Gifu, Japan

**Keywords:** Subarachnoid hemorrhage, Pulmonary edema, Endothelial glycocalyx

## Abstract

Although neurogenic pulmonary edema (NPE) often occurs after aneurysmal subarachnoid hemorrhage (SAH), the mechanism of NPE progression after SAH remains unclear. This study investigates whether pulmonary endothelial glycocalyx (PEG) impairment accompanies NPE after SAH. Accordingly, SAH was induced by endovascular perforation in male mice. The lung tissues of the mice were removed 24 h after SAH induction. The degree of pulmonary edema and lung injury, and the extent of PEG injury were assessed. Water content of lung tissue by the wet/dry method in the SAH group was significantly increased compared to that in the sham group (81.7 % vs. 78.8 %, *P* < 0.01), which suggested NPE following SAH. Lung injury score by hematoxylin and eosin staining in the SAH group, assessed using a semiquantitative scoring system, was also significantly worse than that in the sham group (7.1 vs. 1.2, *P* < 0.001). Scanning electron microscopy images clearly demonstrated that the moss-like glycocalyx lined the endothelial lumen without any interruption in sham mice, whereas those microstructures were severely devastated in SAH mice. Moreover, the fluorescence intensity of tomato lectin was significantly reduced in SAH mice compared to that in sham mice (13.3 vs. 30.7, *P* < 0.001), thereby indicating the loss of PEG. Our results indicate that PEG, which is essential for regulating vascular permeability, is severely impaired after experimental SAH. Maintaining the integrity of PEG is a promising therapeutic strategy for NPE after SAH.

## Highlights

• SAH worsens lung water content and histopathological findings.
• Shedding of PEG after SAH is associated with the mechanism of pulmonary edema.
• Shedding of PEG was observed using SEM and quantified by tomato lectin staining.

## 1 Introduction

Many pathological conditions are involved in the biological reactions after aneurysmal subarachnoid hemorrhage (SAH). In addition to early brain injury and delayed cerebral ischemia, systemic reactions such as neurogenic pulmonary edema (NPE) and Takotsubo cardiomyopathy sometimes occur. The incidence rate of NPE after SAH has been reported to range from 2 to 31 % [[1], [2], [3]], and NPE is more likely to occur in conjunction with severe clinical and radiographic presentations, aneurysms located in the posterior circulation, and advancing age [1,[3], [4], [5], [6]]. Although there are a few epidemiological reports, the reason why pulmonary edema occurs after SAH is completely unknown.

Advances in electron microscope technology have provided the observation and understanding finer structures of the vascular endothelium. The inner surface of healthy vascular endothelial cells is covered with a polysaccharide layer called the endothelial glycocalyx, which covers the inside of the vascular lumen as a barrier between blood and tissues, and plays an important role in the physiological function of endothelial cells, including the regulation of vascular permeability [7]. However, it is an unstable and fragile structure that is damaged and disrupted by various stimuli, including the inflammation and systemic stress [8]. Damage to the vascular endothelial glycocalyx leads to increased vascular permeability in target organs.

We hypothesized that the pulmonary endothelial glycocalyx (PEG) is impaired under acute stress conditions following SAH, resulting in pulmonary edema and aimed to investigate the reason pulmonary edema occurs after SAH.

## 2 Materials and methods

### 2.1 Animals

All experiments were performed in accordance with the guideline of the experimental committee of Gifu Pharmaceutical University (Approved No. 2024–040) and the institutional animal research committee of Gifu University (AG–P–N–20240076, Gifu, Japan), and reported in accordance with ARRIVE guidelines (Animal Research: Reporting of In Vivo Experiments) [9]. Male ddY mice (body weight, 30–40 g; aged, 7–8 weeks) were obtained from Japan SLC, Inc. (Hamamatsu, Shizuoka, Japan). All mice were maintained on a 12 h light/dark cycle under conditions of controlled humidity and stable temperature. They were provided with standard laboratory food and filtered clean water ad libitum.

### 2.2 Surgical methods

SAH was induced using the endovascular perforation technique. Mice were anesthetized with 2–3 % isoflurane and maintained with 1–2 % isoflurane in 70 % N_2_O/30 % O_2_ using an animal general anesthesia machine (Soft Lander; Sin-ei Industry Co., Ltd., Saitama, Japan) without intubation, as previously described [10]. After the skin incision of the necks of the mice, the right common carotid artery (CCA), internal carotid artery (ICA), and external carotid artery (ECA) were exposed. A 5-0 blunted nylon monofilament suture was inserted through the right ECA into the ICA under the CCA occlusion, and advanced until a little resistance to perforate the ICA bifurcation. After the perforation and removal of the nylon, the ECA was coagulated and the CCA was re-perfused. In the sham group, the nylon monofilament suture was inserted and then removed without perforation.

### 2.3 Study design

All mice were randomly assigned by drawing lots to either the SAH group or the sham group by a blinded investigator, and mice that died before euthanasia were excluded from the analysis. Their lungs were evaluated 24 h after the procedure. The water content of lungs, histopathology by hematoxylin and eosin (H&E) staining, fluorescent staining using lectin, and scanning electron microscopy (SEM) were assessed in each group.

### 2.4 Neurobehavioral tests and SAH grading

All mice were euthanized after neurobehavioral tests 24 h after the procedure. Assessment of neurological function was performed by two blinded investigators using the modified neurological score (mNS) [11]. The evaluation consisted of the following eight tests, each of which was scored on a scale of 0–2 or 0–3; spontaneous activity, climbing, balance, side stroking, vibrissae touch, visual, forelimb use, and hind limb use. The average scores were calculated, and a lower score indicates more severe neurological deficits.

The SAH grading score was assessed by subarachnoid clot volume of basal cistern [12]. The subarachnoid blood clots are evaluated in 6 segments of basal cistern and assigned a grade from 0 to 3 as follows: 0, no SAH; 1, minimal SAH; 2, moderate SAH with recognizable cerebral arteries; 3, SAH obliterated the cerebral arteries. Total scores from all 6 segments were evaluated, and a higher score indicates more severe grade.

### 2.5 Evaluation of pulmonary edema

Pulmonary edema was assessed by measuring the water content of lungs. After the lungs of mice were sharply dissected and removed, each tissue was immediately weighted (wet weight) and placed in an 80 °C oven for 24 h. Each dried tissue was weighed again (dry weight), and wet – dry weight/wet weight was calculated as tissue water content [13].

### 2.6 Histopathological findings

A separate set of mice, distinct from those used for the water content measurements, was used for histopathological examination. After the mice were perfused intracardially with 4 % paraformaldehyde, the lungs were removed and soaked in 4 % paraformaldehyde for 24 h at 4 °C. After deparaffinization of each tissue, sections were cut into 5-μm-thick slices [14]. The slides, stained with H&E, were assessed by using a microscope for histopathological analyses. Three blinded investigators calculated the lung injury score using a semiquantitative scoring system, as the previously described [15]. Three visual fields were randomly selected for each tissue section. Inflammation, edema, hemorrhage, and alveolar septal thickening were each scored from 0 to 4 point. Edema and hemorrhage were divided into the following classification: absent (0 points), mild (<10 % alveoli involved; 1 point), moderate (10–30 % alveoli involved; 2 points), severe (30–50 % alveoli involved; 3 points), or very severe (>50 % alveoli involved; 4 points). Alveolar septal thickening was measured as the vertical distance at the thickest part and divided into the following classifications: absent (thickness <15 μm; 0 points), mild (thickness in 15–30 μm; 1 point), moderate (thickness in 30–45 μm; 2 points), severe (thickness in 45–60 μm; 3 points), or very severe (thickness >60 μm; 4 points). Inflammation was evaluated by counting the number of inflammatory cells/× 100 field. The total scores were averaged for each parameter and section. Furthermore, the scores were averaged for each investigator.

### 2.7 Lectin staining intensity

To quantitatively analyze the degree of PEG injury, the fluorescence intensity by the *Lycopersicon esculentum* lectin staining was measured. Approximately, 100 μL of 50 % tomato lectin (DL-1177-1; Vector Laboratories, Burlingame, CA, United States) dissolved in 1 % PBS was injected into the jugular vein 10 min before sacrificing without perfusion for fixation [16]. Lungs were embedded in an optimal cutting temperature compound and frozen by liquid nitrogen. Frozen blocks were cut into 10-μm-thick slices using a cryostat. The intensity of tomato lectin was measured using a confocal fluorescence microscope (BZ-X810; Keyence, Osaka, Japan) and scored by ImageJ software version 1.54G. The intensity was evaluated by averaging the values from 10 fields per mouse in the focal plane, for a total of n = 6, as previously described [16].

### 2.8 Scanning electron microscopy

The PEG was visually observed using a scanning electron microscopy (SEM, S-4500; Hitachi, Tokyo, Japan), as previously described [17], and compared between the sham and SAH model mice. The mice were perfused intracardially with a solution composed of 2 % glutaraldehyde, 2 % sucrose, 0.1 M sodium cacodylate buffer, and 2 % lanthanum nitrate. After the lungs were removed and diced, three or four pieces of approximately 1 mm^3^ each were soaked in the perfusion solution for 2 h for fixation and overnight in a solution without glutaraldehyde. The samples were washed in alkaline sucrose (2 %) solution and dehydrated through a graded ethanol series. Each sample was placed on an iron plate chilled with liquid nitrogen and sprinkled with ethanol. After the samples were frozen, they were fractured using a chisel such that they were not touched directly. The samples were incubated in *tert*-butyl alcohol at room temperature. After the *tert*-butyl alcohol solidified, it was freeze-dried and the samples were observed using SEM.

### 2.9 Statistical analysis

All statistical analyses were performed by blinded investigators using the R statistical package (version 4.2.1; R Foundation for Statistical Computing, Vienna, Austria). The Shapiro-Wilk test was used to confirm normality of the data, and the Student's *t*-test was performed for comparison between the sham and SAH groups. A one-way analysis of variance (ANOVA) was performed to compare the time course of lung water content, and the Holm-Bonferroni method was used to adjust the P-values for multiple comparisons (Supplementary Fig. 1A). Data are presented as mean ± standard error of the mean. Statistical significance was set at *P* < 0.05.

## 3 Results

### 3.1 SAH worsens lung water content and histopathological findings

Six mice in the SAH group died after SAH induction and were excluded from the analysis, resulting in a mortality rate of 16.2 %.

The water content of lungs was assessed 6, 24, and, 48 h after SAH induction. The water content was highest after 24 h, and no significant difference was observed between the 48 h and sham groups (Supplementary Fig. 1A). Therefore, the lungs were evaluated 24 h after SAH induction. There was no difference in the water content between the naïve and sham groups (Supplementary Fig. 1B). Brain images of the sham and SAH model mice are shown in Fig. 1A. The mNS of the SAH group was significantly lower and SAH grading score of the SAH group was significantly higher than those of the sham group (*P* < 0.001; Fig. 1B and C). Assessment of the pulmonary edema is shown in Fig. 1D. In the SAH group, the water content of the lungs was significantly higher than that in the sham group (81.7 % vs 78.8 %; vs. sham, *P* < 0.01, each group; n = 6). Representative lung images of histopathological findings acquired from H&E staining are shown in Fig. 1E. While the sections from the sham group showed almost normal structure, those from the SAH group showed histopathological changes involving interstitial edema, hemorrhage, alveolar septal thickening, and neutrophil infiltration. In the SAH group, lung injury score was significantly higher than that in the sham group (7.1 vs 1.2; vs. sham, *P* < 0.001, each group; n = 6; Fig. 1F and Supplementary Fig. 2).

**Fig. 1:**
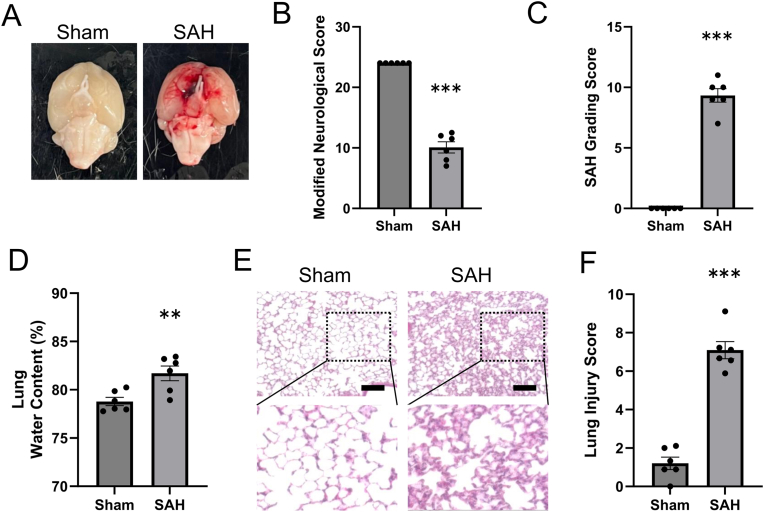
SAH worsens lung water content and lung injury score. **A.** Representative images showing blood clots around the circle of Willis. **B.** Total mNS score. The mNS in the SAH group was significantly lower than that in the sham group. Each group; n = 6. (*P* < 0.001, 95 % confidence interval [CI]: 11.85–15.98) **C.** SAH grading score. The score in the SAH group was significantly higher than that in the sham group. Each group; n = 6. (*P* < 0.001, 95 % CI: −10.58 to −8.09) **D.** Quantification of the lung water content. The lung water content in the SAH group was significantly higher than that in the sham group. Each group; n = 6. (*P* = 0.007, 95 % CI: −4.86 to −0.97) **E.** Representative images acquired from hematoxylin and eosin staining of lungs 24 h after SAH. Scale bar = 100 μm. **F.** Histopathological scores of lung injury. The lung injury score in the SAH group was significantly higher than that in the sham group. Each group; n = 6. (*P* < 0.001, 95 % CI: −7.12 to −4.66) mNS; modified neurological score, SAH; subarachnoid hemorrhage. ∗∗*P* < 0.01 versus sham group. ∗∗∗*P* < 0.001 versus sham group.

### 3.2 SAH damaged the PEG

To visualize the ultrastructure of the PEG, SEM using lanthanum nitrate staining was performed. The PEG in both sham and SAH model mice is shown in Fig. 2. SEM clarified that the moss-like glycocalyx lined the endothelial lumen without any interruption in the sham mice. However, the PEG in the SAH model mice disrupted and fell off.

**Fig. 2:**
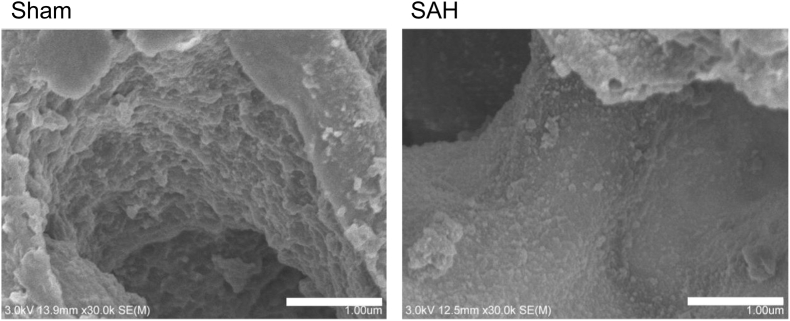
SAH damages pulmonary vascular endothelial glycocalyx. Representative images acquired from SEM. In the sham mice, the moss-like glycocalyx lined the endothelial lumen without any interruption. In the SAH model mice, the PEG disrupted and fell off. Scale bar = 1 μm. Each group; n = 1. SEM; scanning electron microscopy, SAH; subarachnoid hemorrhage, PEG; pulmonary endothelial glycocalyx.

**Fig. 3:**
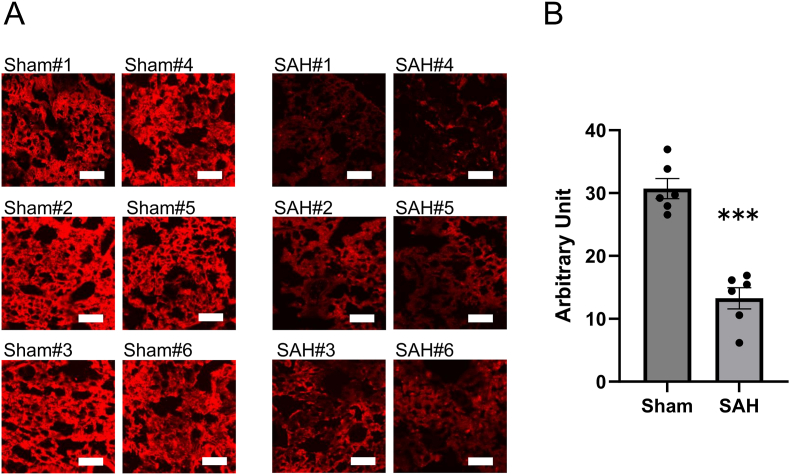
SAH reduces lung area stained with tomato lectin. **A.** Representative images acquired from fluorescence staining using tomato lectin. In the sham group, many areas stained with tomato lectin remained, whereas in the SAH group, these areas were clearly reduced. A separate set of mice was used between each group. Scale bar = 100 μm. **B.** Fluorescence intensity score of tomato lectin in the SAH group was significantly lower than that in the sham group. Ten fields per mouse were averaged; the statistical unit is the mouse (n = 6/group, *P* < 0.001, 95 % CI: 12.27–22.61) SAH; subarachnoid hemorrhage. ∗∗∗*P* < 0.001 versus sham group.

We performed intensity measurements using tomato lectin staining, which enabled quantitative assessment of the extent of the PEG injury. Findings of tomato lectin staining are shown in Fig. 3A. In the sham group, many areas stained with tomato lectin remained, whereas in the SAH group, these areas were clearly reduced. In the SAH group, intensity score of tomato lectin in the PEG was significantly lower than that in the sham group (13.3 vs 30.7; vs. sham, *P* < 0.001, each group; n = 6; Fig. 3B). The PEG was clearly damaged by the occurrence of SAH.

## 4 Discussion

Although NPE is a well-known systemic reaction following aneurysmal SAH, the detailed mechanisms remain unclear. It is believed that the autonomic nervous system response to elevated intracranial pressure plays an important role in the pathogenesis of NPE [4]. However, what actually happens at the level of pulmonary vascular endothelial cells remains unknown. Therefore, we focused on the PEG to investigate at a microscopic level the reason pulmonary edema occurs after SAH.

The endothelial glycocalyx has been difficult to observe and its three-dimensional structure has been difficult to understand because of its instability and fragility. Recent technological advances such as lanthanum nitrate staining with more careful perfusion method has enabled visualization of these fragile structures [17]. This has revealed that the vascular endothelial glycocalyx is impaired in acute conditions such as sepsis [18], trauma [19], respiratory distress syndrome [20], ischemia-reperfusion injury [21], and burns [22], and in chronic conditions such as diabetes mellitus [23], hyperlipidemia [24], chronic kidney disease [25], and aging [26]. Our experiments reveal that in addition to these pathological conditions, the PEG is also impaired after aneurysmal SAH. This result makes sense, considering the fragility of the endothelial glycocalyx, which is detached by various stimuli.

The endothelial glycocalyx has various important functions, such as maintenance of smooth blood flow [27], the regulation of vascular permeability [7], inhibition of blood coagulation [28], and inhibition of leukocyte adhesion [29]. From the perspective of interstitial fluid retention, the regulation of vascular permeability seems to be a particularly important function of the vascular endothelial glycocalyx. It also has a high reflection capability for albumin because the glycosaminoglycan, one of its components, has the high density of negative electric charges [18]. One of the roles of endothelial glycocalyx is to maintain the intravascular osmotic pressure by retaining albumin within the blood vessels. Damage to the PEG following SAH makes it impossible to maintain intravascular osmotic pressure, increases pulmonary vascular permeability, and induces interstitial fluid retention. As the endothelial glycocalyx is located on the luminal side of the vascular endothelial cells, it is the fine structure that shows early damage, and this is a potentially important mechanism of pulmonary edema.

In addition, differences in the properties of the endothelial glycocalyx depending each organ may explain the organ-specific susceptibility to fluid retention in the lungs after SAH [17]. The PEG is a thin layer, and transmission electron microscopy revealed that the percentage of endothelial glycocalyx area in the lungs was 3.7 % [8]. This is significantly thinner than that of endothelial glycocalyx area in the heart and brain, and is thought to be due to the need for gas exchange between the capillaries and alveoli in the lungs. Therefore, this thinness of the PEG is efficient under normal conditions. However, under the stress of SAH, this may affect glycocalyx shedding and be related to lung-specific increased permeability.

To our knowledge, no basic experiments have been conducted to determine whether pulmonary vascular endothelial cells or alveolar epithelial cells are damaged after SAH. However, both cells are damaged after ischemic stroke, which is similar to the characteristics of increased permeability pulmonary edema, including vascular endothelial barrier disruption and alveolar epithelial damage, as exemplified by acute respiratory distress syndrome [30]. This study focused on the vascular endothelial glycocalyx and did not investigate alveolar epithelial cells. The extent to which cell damage in NPE after SAH affects the alveolar structure is important for a more detailed understanding of the pathogenesis.

Despite these reasonable results, this study has limitations. First, the study revealed that damage to the PEG is involved in NPE following SAH. However, the study did not clarify the accurate mechanism by which the stress under SAH impairs the glycocalyx. It is speculated that catecholamine surges and inflammation are involved in the shedding of the PEG; therefore, further research is needed. Furthermore, differences in the degree of glycocalyx shedding depending on the severity of SAH and its relationship to NPE are important topics. However, owing to the small sample size, this study was unable to investigate the relationship between SAH severity and NPE. Moreover, although this study investigated the relationship between NPE following SAH and the PEG, the detrimental effects of NPE on the clinical condition of mice and cerebral blood flow were not assessed. Additionally, the mice were not intubated, which may have affected blood gases and tissue oxygenation. Therefore, future experiments incorporating more accurate clinical conditions are needed. Furthermore, the area most susceptible to damage is an important consideration; however, this study did not provide data on whether shedding of PEG is localized or global. Additionally, this study did not investigate the glycocalyx pathway, upstream drivers, nor the detailed mechanism of pulmonary edema. Therefore, further research is needed to clarify the direct mechanism. Finally, although only a limited number of mice exhibit neurogenic stress cardiomyopathy [31], this study data may be contaminated by pulmonary edema due to catecholamine-induced cardiac dysfunction. Accordingly, future experimental protocols that simultaneously examine not only the lungs but also the heart may be necessary.

The study results suggest that the underlying mechanism of NPE following SAH, which has not previously been clearly elucidated, involves impairment of the PEG. The study serves as a reference point for future research and provides valuable insights into understanding the pathology of the condition and managing it.

## Ethics approval

All experiments were performed in accordance with the guidelines of the experimental committee of Gifu Pharmaceutical University (Approved No. 2024–040) and the institutional animal research committee of Gifu University (AG–P–N–20240076, Gifu, Japan).

## Funding

This study was supported by 10.13039/501100001691Japan Society fot the Promotion of Science (JSPS) KAKENHI (25 K12314).

## CRediT authorship contribution statement

**Nozomi Sasaki:** Conceptualization, Data curation, Formal analysis, Funding acquisition, Investigation, Methodology, Visualization, Writing – original draft. **Yusuke Egashira:** Conceptualization, Project administration, Supervision, Visualization, Writing – review & editing. **Hideshi Okada:** Investigation, Methodology, Resources, Supervision, Writing – review & editing. **Chihiro Takada:** Investigation, Methodology, Resources. **Shinomi Sasaibe:** Data curation, Formal analysis, Investigation. **Masaki Kumagai:** Data curation, Formal analysis, Investigation. **Yoshiki Kuse:** Formal analysis, Resources, Writing – review & editing. **Shinsuke Nakamura:** Resources, Writing – review & editing. **Hirofumi Matsubara:** Writing – review & editing. **Yukiko Enomoto:** Writing – review & editing. **Toru Iwama:** Supervision, Writing – review & editing. **Tsuyoshi Izumo:** Project administration, Supervision, Writing – review & editing. **Hideaki Hara:** Funding acquisition, Project administration, Resources, Supervision, Writing – review & editing. **Masamitsu Shimazawa:** Funding acquisition, Project administration, Resources, Supervision, Writing – review & editing.

## Declaration of competing interest

The authors declare the following financial interests/personal relationships which may be considered as potential competing interests:Nozomi Sasaki reports financial support was provided by JSPS KAKENHI. If there are other authors, they declare that they have no known competing financial interests or personal relationships that could have appeared to influence the work reported in this paper.

## Figures and Tables

**figs1:**
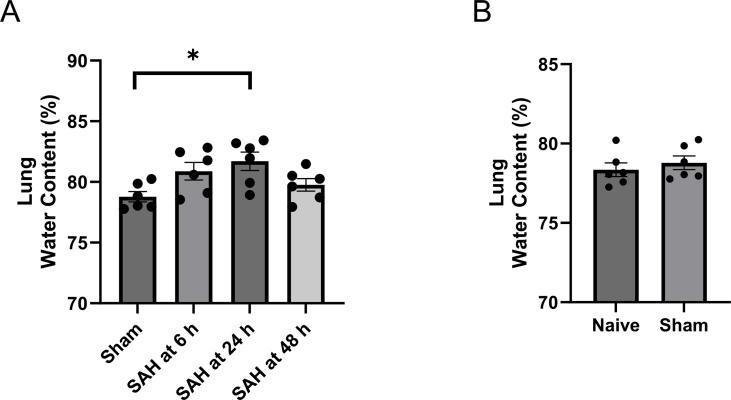


**figs2:**
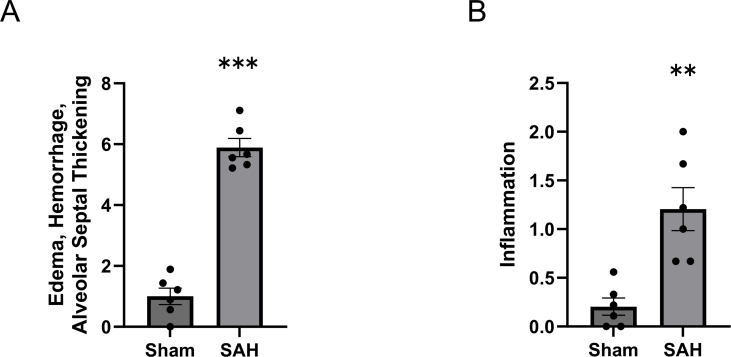


## Data Availability

Data will be made available on request.
