# Supplementary material for: Pulmonary edema following subarachnoid hemorrhage is associated with impairment of pulmonary vascular endothelial glycocalyx
Source: Biochem Biophys Rep. 2025 Dec 18;45:102420. doi: 10.1016/j.bbrep.2025.102420 (PMC12794039; doi:10.1016/j.bbrep.2025.102420)
Supplement: Multimedia component 1 [file mmc1.docx]

**Supplementary Figure 1.** **Lung water content increases significantly 24 h after SAH induction.**

**A.** The water content of the lungs 24 h after SAH was significantly higher than that in the sham group (*P* = 0.021, 95% CI: −5.39 to −0.45, vs. sham), and no significant difference was observed after 48 h compared with the sham group (*P* = 0.651, 95% CI: −3.44 to 1.5, vs. sham, n = 6 for each group). **B.** No significant difference was observed between the naïve and sham groups in terms of water content (*P* = 0.492, 95% CI: −1.79 to 0.92, vs. sham, n = 6 for each group).

SAH; subarachnoid hemorrhage

* *P* < 0.05 versus sham group.

**Supplementary Figure 2. Lung injury score quantified separately for lung injury and inflammation.**

**A.** Edema, hemorrhage, and alveolar septal thickening in the SAH group was significantly higher than that in the sham group (n = 6 for each group, *P* < 0.001, 95% CI: −5.79 to −3.98). **B.** Inflammation in the SAH group was significantly higher than that in the sham group (n = 6 for each group, *P* = 0.002, 95% CI: −1.53 to −0.47)

SAH; subarachnoid hemorrhage.

** *P* < 0.01 versus sham group. *** *P* < 0.001 versus sham group.
