# Supplementary material for: Pulmonary edema following subarachnoid hemorrhage is associated with impairment of pulmonary vascular endothelial glycocalyx
Source: Biochem Biophys Rep. 2025 Dec 18;45:102420. doi: 10.1016/j.bbrep.2025.102420 (PMC12794039; doi:10.1016/j.bbrep.2025.102420)
Supplement: Multimedia component 2 [file mmc2.docx]

**Supplementary Table 1. Modified neurological score, SAH grading score, and lung water content of both groups**

| **Sham group** | **mNS** | **SAH grading score** | **Lung water content (%)** |
| --- | --- | --- | --- |
| Sham mouse 1 | 24 | 0 | 78.04 |
| Sham mouse 2 | 24 | 0 | 78.84 |
| Sham mouse 3 | 24 | 0 | 77.77 |
| Sham mouse 4 | 24 | 0 | 79.86 |
| Sham mouse 5 | 24 | 0 | 80.24 |
| Sham mouse 6 | 24 | 0 | 77.95 |
| **SAH group** | **mNS** | **SAH grading score** | **Lung water content (%)** |
| SAH mouse 1 | 11.5 | 9 | 81.95 |
| SAH mouse 2 | 9.5 | 7 | 78.93 |
| SAH mouse 3 | 12 | 9 | 79.93 |
| SAH mouse 4 | 12.5 | 11 | 82.77 |
| SAH mouse 5 | 7 | 10 | 83.43 |
| SAH mouse 6 | 8 | 10 | 83.19 |

mNS, modified neurological score; SAH, subarachnoid hemorrhage

**Supplementary Table 2. Lung injury score of both groups**

| **Sham group** | **Edema, Hemorrhage,  Alveolar septal thickening** | **Inflammation** | **LIS** |
| --- | --- | --- | --- |
| Sham mouse 1 | 1.89 | 0.22 | 2.11 |
| Sham mouse 2 | 1.22 | 0.11 | 1.33 |
| Sham mouse 3 | 0 | 0 | 0 |
| Sham mouse 4 | 1.44 | 0.56 | 2 |
| Sham mouse 5 | 0.56 | 0.33 | 0.89 |
| Sham mouse 6 | 0.89 | 0 | 0.89 |
| **SAH group** | **Edema, Hemorrhage,  Alveolar septal thickening** | **Inflammation** | **LIS** |
| SAH mouse 1 | 5.56 | 1.67 | 7.22 |
| SAH mouse 2 | 5.67 | 1 | 6.67 |
| SAH mouse 3 | 5.22 | 0.67 | 5.89 |
| SAH mouse 4 | 6.44 | 0.67 | 7.11 |
| SAH mouse 5 | 7.11 | 2 | 9.11 |
| SAH mouse 6 | 5.33 | 1.22 | 6.56 |

SAH, subarachnoid hemorrhage; LIS, lung injury score

**Supplementary Table 3. Lectin staining intensity of both groups**

| **Sham group** | **Lectin intensity (Arbitary unit)** |
| --- | --- |
| Sham mouse 1 | 27.96 |
| Sham mouse 2 | 26.56 |
| Sham mouse 3 | 29.73 |
| Sham mouse 4 | 29.21 |
| Sham mouse 5 | 33.85 |
| Sham mouse 6 | 36.95 |
| **SAH group** | **Lectin intensity (Arbitary unit)** |
| SAH mouse 1 | 10.58 |
| SAH mouse 2 | 6.18 |
| SAH mouse 3 | 14.42 |
| SAH mouse 4 | 15.43 |
| SAH mouse 5 | 16.88 |
| SAH mouse 6 | 16.15 |

SAH, subarachnoid hemorrhage
